# Molecular and phylogenetic characterization of *Thelohanellus boggoti* Qadri, 1962 (Cnidaria, Myxosporea, Bivalvulida) infecting the fin of Indian minor carp *Labeo dero* (Hamilton, 1822)

**Published:** 2017-03

**Authors:** Harpreet Kaur, Aditya Gupta

**Affiliations:** 1 Department of Zoology, Panjab University, Chandigarh-160014, Chandigarh, India; 2 Department of Zoology and Environmental Sciences, Punjabi University, Patiala-147002, Punjab, India

**Keywords:** *Thelohanellus boggoti*, *Phylogeny*, *Labeo dero*, 18S rDNA, Ranjit Sagar wetland

## Abstract

A myxozoan parasite belonging to the genus *Thelohanellus *Kudo, 1933 (Cnidaria, Myxosporea, Bivalvulida) was isolated from the fins of *Labeo dero *inhabiting Ranjit Sagar Wetland, Punjab, India. The plasmodium was 0.5-0.7 mm in diameter each containing 80- 100 number of myxospores. Myxospores were egg shaped to ovoidal in valvular view having bluntly pointed anterior and broad rounded posterior end measuring 8.36x 4.77 µm. Polar capsule was single, broadly pyriform in shape, measuring 4.77x3.98 µm in size containing a polar filament coiled perpendicular to the longitudinal axis of myxospore body making 7-9 turns. Blast analysis of 18S rDNA sequence of the isolate demonstrated 98% homogeneity with *Thelohanellus *sp. (KU155561, Unpublished), followed by 91% with *T. caudatus* (KM252684) and *Thelohanellus *sp. FCO (KR819273). The intensity of infection was recorded to be light as indicated by fin plasmodial index (FPI = 1). In the present study, *T. boggoti* has been described using 18S rRNA gene and phylogenetic analysis using MEGA.6.

## INTRODUCTION

The Myxozoa are important fish parasites characterized by a two host life cycle involving invertebrates and vertebrates as definitive and intermediate hosts, respectively. There are about 2200 described myxosporean species [1] characterised by morphology and morphometrics of the myxospores representing about 18% of cnidarian species.Certain myxozoans have been recorded to cause serious damage to economically important fishes such as salmon and trout in all parts of the globe namely proliferative kidney disease caused by *Tetracapsuloides bryosalmonae* [2], whirling disease by *Myxobolus cerebralis* [3], turbot enteromyxosis by *Enteromyxum scophthalmi* [4] and gill disease by *Thelohanellus bifurcata *[5].

Ranjit Sagar Wetland, also known as the Thein Dam is at the boundary of three states of Punjab, Jammu and Kashmir and Himachal Pradesh, India on river Ravi. It is a manmade, riverine and lacustrine wetland with freshwater ecology. It lies at an altitude of about 540 msl at 32˚ 26΄ 30´´ N Latitude and 75˚ 43΄ 30´´ E Longitude and is spread over an area of 87.60 sq km. It is a cold water wetland inhabiting high diversity of fish species. Members of the genus *Thelohanellus* Kudo, 1933 are characterized by having shell with smooth valves and single polar capsule [6]. So far 108 nominal species have been recorded throughout the world, out of which 32 species have been described from India [7, 8]. The identification of the genus is based on the spores, a propagative life cycle stage enclosed in plasmodium. The authors have been successful in characterizing some of the species of *Thelohanellus* from northern part of India [5, 9-17]. The present report is the first on the molecular and phylogenetic characterizations of *T. boggoti* as molecular characterizations of already described *Thelohanellus* species have been done by earlier workers [18, 19].

## MATERIALS AND METHODS

**Collection and Microscopy:** Specimens of *Labeo dero *(n= 50) with average length of 15-20 cm were procured from the various catchment sites of Ranjit Sagar Wetland. They were anesthetized with chloroform at a concentration of 40 mg/l, and necropsied to study parasites using a stereo microscope. After necropsy, plasmodia present on the fins were removed, teased on a slide and examined under phase contrast microscope (Magnus MLX) and myxospore morphology was studied, measured and photographed. The parasite frequency index (PFI) was found to be 20% (10/50). Fin plasmodial index (FPI) was calculated on the basis of number of plasmodia present per fin visible under the stereozoom binocular microscope and with the naked eye [20] using the following scale: no plasmodium (no infection-0); 1-5 plasmodia (light infection-1); 5-10 plasmodia (moderate infection-2); 10-20 plasmodia (heavy infection-3); 20-50 plasmodia or more (severe infection-4).

**DNA extraction, PCR amplification and Sequencing:** The myxospores were collected from the infected fish (10 in number) and fixed in absolute alcohol for molecular and phylogenetic analysis. The parasite DNA was extracted using the DNeasy Blood and Tissue Kit (Qiagen) following the manufacturer’s instructions. The product was then quantified in a Nanodrop (Thermo Scientific, Wilmington, USA) spectrophotometer at 160 ng/μl. The specific primers for 18S region My1F (5’-CTA ATC CCG GTA ACG AAC GA-3’) and My10R (5’-CGT CCT CGC AAC AAA CTG TA-3’) standardized during the present study were used for the amplification of 18S rDNA using Eppendorf Master Cycler Pro S. The PCR was carried out, according to [21] at the final volume of 25 μl using the primers which successfully amplified the fragments of 818 bp of the 18S rDNA gene. The amplification reactions were conducted with 50 ng of genomic DNA, 12.5 μl of 1× reaction buffer (HiMedia), 1.0 μl of each primers, 1.0 μl of total DNA and 10.5 μl of nuclease free water. Amplification was done by initial denaturation at 95°C for 3 min, followed by 34 cycles of denaturation at 95°C for 30 s, annealing of primers at 57°C for 30 s, extension at 72°C for 1 min 20 s. The final extension was at 72°C for 10 min. The PCR product was analyzed on a 2% agarose gel containing 0.5 μg/ml ethidium bromide in 1× Tris-acetate-EDTA (TAE) buffer and size was estimated by comparison with the 100 bp Plus DNA Ladder. The amplified product was commercially sequenced at Molecular Diagnostic and Research Laboratories, Chandigarh (India). 

**Phylogenetic analysis:** The phylogenetic analysis was done on a selection of 18S rDNA sequences that comprised the new sequence (KU884967) and 25 additional sequences from closely related sequences showing 86% homogeneity or above in NCBI GenBank database using the basic local alignment tool [22]. *Sphaerospora molnari* (AF378345) isolated from *Carassius auratus* was taken as an outgroup. Genetic distance analyses were conducted using the Kimura 2-parameter model [13] in MEGA6 software [23]. Included codon positions were 1st + 2nd + 3rd + Noncoding. All positions containing gaps and missing data were eliminated. Sequence alignment was performed by Multiple Sequence Comparison by Log-Expectation (MUSCLE). The best fit substitution model for constructing the phylogenetic tree was K2+G having the lowest Bayesian score of 6481.279. The tree was generated using Neighbour- Joining having 1000 bootstrap values and was proportional to the number of substitutions per site. Tajima’s neutrality values were calculated by selecting all the species. All the analysis was conducted in MEGA6. The results were exported and copied into excel files and saved as tables.

## RESULTS AND DISCUSSION

**Figure 1:**
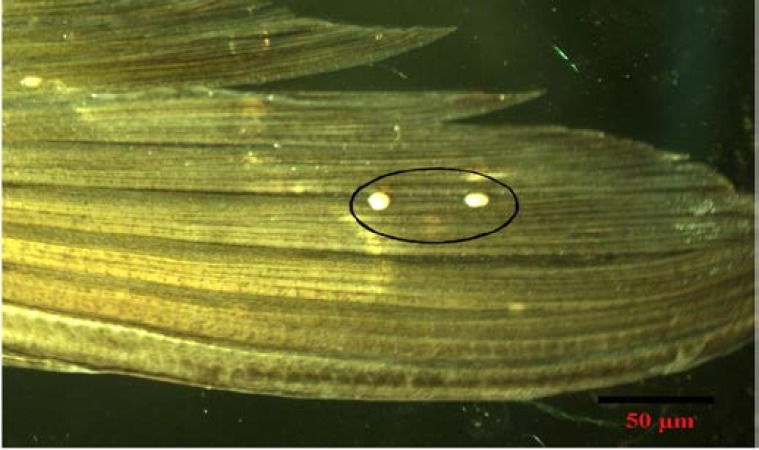
Photomicrograph of infected fin of Labeo dero showing plasmodia of Thelohanellus boggoti.

Plasmodia were minute, round to irregular, creamish-white which measured 0.5- 0.7 mm in diameter. They were found attached to the fins and were histozoic. There were 2-5 pasmodia per fin having 80-100 myxospores per plasmodium. Clinical signs on the fin were apparent showing pale spots (Fig. 1).

**Figure 2:**
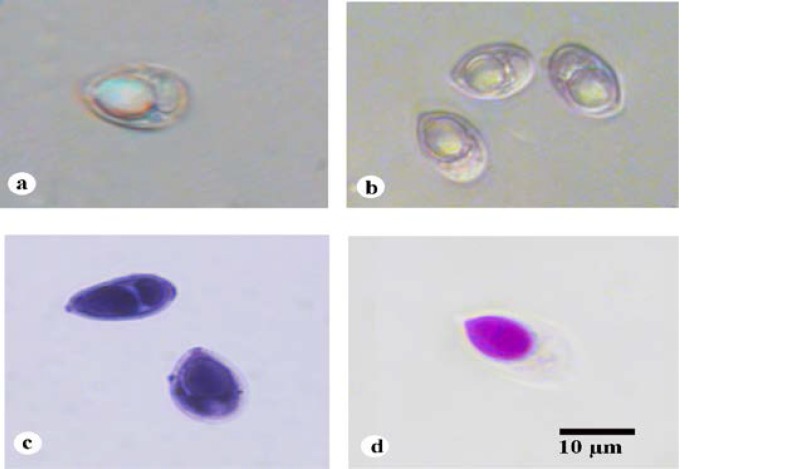
Photomicrograph of myxospores of Thelohanellus boggoti from the fin of Labeo dero. (a) Fresh myxospore (dark field microscopy); (b) Fresh myxospores (phase contrast microscopy); (c) Myxospores stained in Iron-Haematoxylin; (d) Myxospore stained in Ziehl-Neelsen

Myxospores measured 8.36x4.77µm, egg shaped to ovoidal in valvular view having bluntly pointed anterior end and broad rounded posterior end. Shell valves were thick, smooth, symmetrical and measured 0.66µm in thickness. Parietal folds were absent. Polar capsule was flask-shaped with distinct neck measuring 4.77*3.98µm and occupied more than half of the myxospore body cavity. Polar filament formed 7-9 coils and was arranged perpendicular to the polar capsule axis. One capsulogenic nucleus was present beneath the polar capsule measuring 1.4µm in diameter. Sporoplasm was agranular, homogenous, moon-shaped which occupied whole of the extracapsular space behind the polar capsule. Sporoplasm contained two sporoplasmic nuclei and an iodinophilous vacuole was also present which measured 1.25x1.35µm and 1.4x1.35μm in diameter respectively (Fig. 2, Table 1).

Type-host: *Labeo dero* vern dero, (Family: Cyprinidae) Type-locality: Ranjit Sagar wetland, Punjab, India. Site of infection: Fins. Type materials: Slide no. M/ZN/16.12.2014 and M/IH/16.12.2014, Parasitology Laboratory, Department of Zoology and Evironmental Sciences, Punjabi University, Patiala (India). Parasite frequency index (PFI): 20% (10/50) Fin plasmodial index (FPI): 1 (2-5 plasmodia per fin) indicating light infection.

**Table 1:** Measurements (µm) and ratio of *Thelohanellus boggoti *Qadri, 1962

**Characters**	**Range**	**Mean Values**	**SD**	**CV**
LS	8.30-8.42	8.36	0.08	0.007
WS	4.70-4.84	4.77	0.09	0.009
LPC	4.72-4.82	4.77	0.07	0.005
WPC	3.95-4.01	3.98	0.04	0.001
Ratio: LS/WS		1.75		
NC		7-9		
Parietal Folds		Absent		

(LS length of spore, WS width of spore, LPC length of polar capsule, WPC width of polar capsule, NC number of coils, SD standard deviation, CV coefficient of variance).

The present observations on *T. boggoti *Qadri, 1962 are in conformity with the original description (LS/WS: 1.70 vs 1.75) except some variations in the size of the myxospore (as indicated by LS/WS ratio) and polar capsule. Earlier, this parasite was recorded from the gills of *Labeo boggot *(Sykes, 1839) in Andhra Pradesh, India*. *Subsequently, it was recorded from the gills of *Catla catla* in Harike wetland, Punjab, India. In the present study, a new host- *Labeo dero *(Hamilton, 1822), a new locality-Ranjit Sagar wetland, Punjab, India and a new site of infection-fins have been recorded for this parasite (Table 2).

**Table 2:** Comparative description of *Thelohanellus boggoti *Qadri, 1962 with original species (measurements in micrometer)

**Species**	**Host**	**Site of infection**	**Locality**	**Spore**	**Polar capsule**
***T. boggoti*** (present study)	*Labeo dero*	Fins	Ranjit Sagar wetland, Punjab (India)	8.36*4.77	4.77*3.98
***T. boggoti*** Qadri, 1962	*Labeo boggot*	Gills	Andhra Pradesh (India)	11.5*6.75	6.2*3.80

The primer sets My1F and MY10R successfully amplified the fragments of 818 bp of the 18S rDNA gene. The edited nucleotide sequence obtained from myxospores of *T. boggoti* was deposited in the GenBank under the accession number of KU884967. The BLAST analysis of *T. boggoti* showed similarity with many Indian species, maximum homogeneity with *Thelohanellus *sp. (KU516661; 98% similarity over 1430 bp) infecting the gills of *L. dero*, *T. caudatus * (KM252684; 91% similarity over 1083 bp) infecting the anal fin of *L. rohita *and *Thelohanellus *sp. FCO (KR819273; 91% similarity over 1066 bp) infecting the operculum and skin of *C. catla* and *T. habibpuri* (KM252681; 90% similarity over 1064 bp) infecting the pelvic fin of *L. rohita* (Table 3). The phylogenetic tree based on the final edited alignment with Neighbour- Joining showed *T. boggoti* in a separate clade with a bootstrap value of 89 comprising *T. habibpuri*, *Thelohanellus *sp. CTPv, *T. caudatus*, *Thelohanellus* sp. FCO and *Thelohanellus* sp. (maximum bootstrap value of 100).

**Figure 3:**
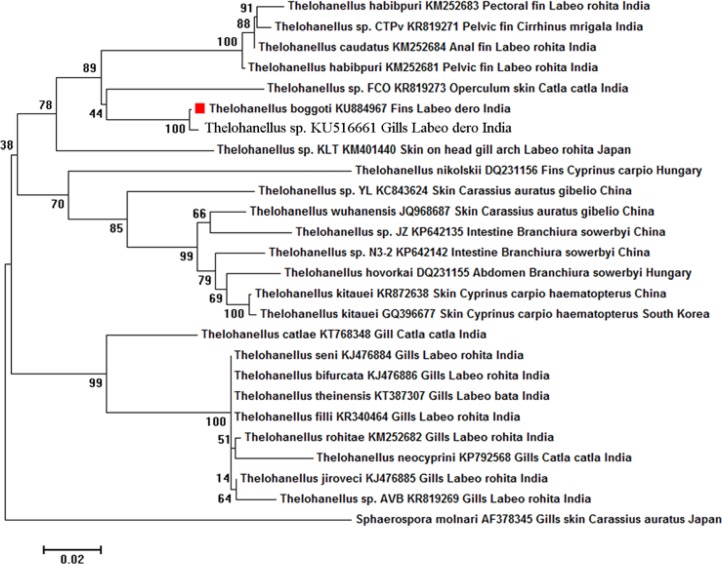
Phylogenetic tree generated by Neighbour-Joining showing the phylogenetic position of Thelohanellus boggoti (KU884967) with other myxosporeans. GenBank accession numbers, organ,host and country names are given and number above nodes indicates bootstrap confi-dence values. Sphaerospora molnari was taken as the out-group. Scale bar: amount of inferred evolutionary change along the branch lengths

**Figure 4:**
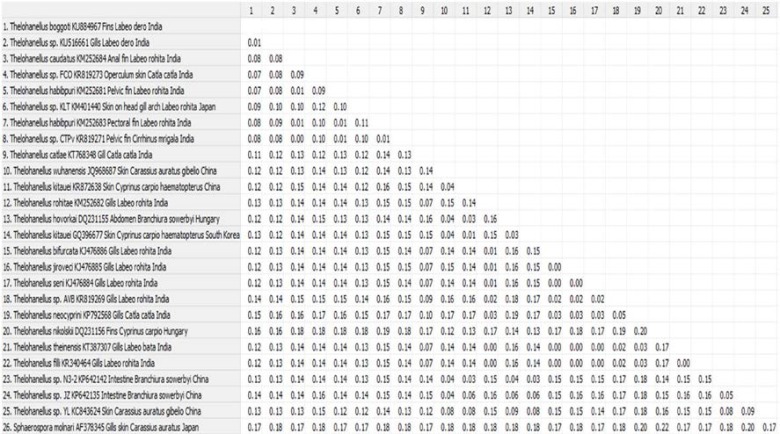
Estimates of evolutionary divergence between the sequences of Thelohanellus boggoti (KU884967) and other myxosporeans available in NCBI

The other clade comprised of all the Indian Thelohanelloid species from gills with a highest bootstrap value of 99. The out-group *Sphaerospora* *molnari* was phylogenetically clustered distinctly as a separate lineage (Fig. 3). Moreover, estimates of evolutionary pair- wise divergence among the sequences of *T. habibpuri*, *Thelohanellus* sp. CTPv, *T. caudatus*, *Thelohanellus* sp. FCO and *Thelohanellus* sp. was 0.09, 0.01, 0.08, 0.09 and 0.01 respectively (Fig. 4). 

The nucleotide frequencies were 26.66% (A), 25.26% (T/U), 20.13% (C) and 27.94% (G). The transition/transversion rate ratios are K1= 3.081 (purines) and K2= 4.819 (pyrimidines). The Gamma distribution among 5 categories was 0.7349. All positions containing gaps and missing data were eliminated. Tajima’s neutrality test for the nucleotide mutation was also done. The D value was found to be 1.16889 meaning some of the alleles were present at low frequencies indicating low genetic diversity among myxosporeans. The present report is the first on the molecular and phylogenetic characterizations of *T. boggoti*.

**Table 3:** Homogeneity of 18S rRNA gene sequences of *T. boggoti* Qadri, 1962 (Accession number KU884967) and other myxosporeans available in NCBI GenBank

**Myxozoan**	**Accession number**	**Organ infected**	** Host **	**Country**	** Query** ** cover**	**Homogeneity (%) to ** ***T. boggoti *** **(KU884967)**
*Thelohanellus *sp.	KU516661	Gills	*Labeo dero *	India	99	1430/1430 (98)
*Thelo T. caudatus*	KM252684	Anal fin	*Labeo rohita *	India	99	1083/1083 (91)
*Thelohanellus *sp. FCO	KR819273	Operculum, skin	*Catla catla *	India	96	1066/1066 (91)
*T. habibpuri*	KM252681	Pelvic fin	*Labeo rohita *	India	99	1064/1064 (90)
* Thelohanellus *sp. KLT	KM401440	Skin on head, gill arch	*Labeo rohita *	Japan	96	1059/1059 (91)
* T. habibpuri*	KM252683	Pectoral fin	*Labeo rohita *	India	99	1051/1051 (93)
* Thel Thelohanellus *sp. CTPv	KR819271	Pelvic fin	*Cirrhinus mrigala *	India	99	1014/1014 (90)
* T. catlae*	KT768348	Gill	*Catla catla *	India	95	985/985 (89)
*T. wuhanensis*	JQ968687	Skin	*Carassius auratus gibelio *	China	97	983/983 (89)
* Thel T. kitauei*	KR872638	Skin	*Cyprinus carpio haematopterus *	China	96	979/979 (89)
* Thelo T. rohitae*	KM252682	Gills	*Labeo rohita *	India	95	976/976 (89)
*T. hovorkai*	DQ231155	Abdomen	*Branchiura sowerbyi *	Hungary	97	970/970 (89)
*Thel T. kitauei*	GQ396677	Skin	*Cyprinus carpio haematopterus *	South Korea	96	968/968 (89)
*T. bifurcata*	KJ476886	Gills	*Labeo rohita *	India	95	963/963 (89)
*T. jiroveci*	KJ476885	Gills	*Labeo rohita *	India	95	948/948 (89)
*T. seni*	KJ476884	Gills	*Labeo rohita*	India	95	939/939 (89)
*T. *sp. AVB	KR819269	Gills	*Labeo rohita*	India	95	894/894 (87)
*T. neocyprini*	KP792568	Gills	*Catla catla*	India	94	887/887 (88)
*T. nikolskii*	DQ231156	Fins	*Cyprinus carpio*	Hungary	97	874/874 (87)
*T. theinensis*	KT387307	Gills	*Labeo bata*	India	95	841/841 (86)
*T. filli*	KR340464	Gills	*Labeo rohita*	India	95	841/841 (86)
*Thelohanellus *sp. N3-2	KP642142	Intestine	*Branchiura sowerbyi*	China	83	798/798 (88)
*Thelohanellus *sp. JZ	KP642135	Intestine	*Branchiura sowerbyi*	China	83	745/745 (87)
*Thelohanellus *sp YL	KC843624	Skin	*Carassius auratus gibelio*	China	76	684/684 (87)
*Sphaerospora molnari*	AF378345	Gills, skin	*Carassius auratus*	Japan	98	830/830 (86)

**Nomenclatural Acts**: 

This work and the Nomenclatural Acts it contains have been registered in ZooBank. The ZooBank Life Science Identifier (LSID) for this publication is:http://zoobank.org/urn:lsid:zoobank.org:pub:303E2E29-1D21-422A-9F34 20484479D701.
